# Therapeutic and space radiation exposure of mouse brain causes impaired DNA repair response and premature senescence by chronic oxidant production

**DOI:** 10.18632/aging.100587

**Published:** 2013-08-06

**Authors:** Shubhankar Suman, Olga C. Rodriguez, Thomas A. Winters, Albert J. Fornace, Chris Albanese, Kamal Datta

**Affiliations:** ^1^ Department of Biochemistry and Molecular & Cell Biology, Georgetown University Medical Center, Washington, DC 20057, USA; ^2^ Lombardi Comprehensive Cancer Center, Georgetown University, Washington, DC 20057, USA; ^3^ Nuclear Medicine Department, Warren Grant Magnuson Clinical Center, National Institutes of Health, Bethesda, Maryland 20892; ^4^ Center of Excellence In Genomic Medicine Research (CEGMR), King Abdulaziz University, Jeddah, SA

**Keywords:** Radiation, heavy ion radiation, premature aging, senescence, oxidative stress, DNA damage, cerebral cortex, long-term effects

## Abstract

Despite recent epidemiological evidences linking radiation exposure and a number of human ailments including cancer, mechanistic understanding of how radiation inflicts long-term changes in cerebral cortex, which regulates important neuronal functions, remains obscure. The current study dissects molecular events relevant to pathology in cerebral cortex of 6 to 8 weeks old female C57BL/6J mice two and twelve months after exposure to a γ radiation dose (2 Gy) commonly employed in fractionated radiotherapy. For a comparative study, effects of 1.6 Gy heavy ion^56^Fe radiation on cerebral cortex were also investigated, which has implications for space exploration. Radiation exposure was associated with increased chronic oxidative stress, oxidative DNA damage, lipid peroxidation, and apoptosis. These results when considered with decreased cortical thickness, activation of cell-cycle arrest pathway, and inhibition of DNA double strand break repair factors led us to conclude to our knowledge for the first time that radiation caused aging-like pathology in cerebral cortical cells and changes after heavy ion radiation were more pronounced than γ radiation.

## INTRODUCTION

Radiation exposure to normal brain tissue during therapeutic and diagnostic procedures is unavoidable and radiation has been shown to affect brain function [1-3]. Radiation therapy remains the main mode of treatment for both the primary and secondary brain tumors and fractionated radiation therapy commonly uses a 2 Gy daily dose-fraction [1,4]. Although important advances have been made in the field of radiation therapy to make it more focused, radiation exposure to normal brain tissue is inescapable leading to long-term functional deficit such as cognitive, visual, and motor impairments. Among the diagnostic procedures, computerized tomographic (CT) scan due to its multiple exposure sequences exposes tissues to a higher radiation doses per scan than a single exposure procedures such as x-ray chest. A single head and neck CT scan, depending on age, could expose brain to radiation doses between 20 and 100 mGy and with marked increase in radiation based diagnostic procedures [5-7], the cumulative radiation dose to brain due to repeated exposure could be high enough to raise long-term health concern such as functional decline and cancer [8]. Epidemiological studies in atom bomb survivors have shown increased cancer risk after exposure to radiation dose between 50 and 200 mGy and exposure at an early age has the highest risk [9]. Cerebral cortex, the outermost layer of the mammalian brain, regulates important functions such as awareness, motor and sensory functions, memory, language, and visual perceptions through its connections to various sub-cortical structures such as the thalamus and basal ganglia. Therefore, any perturbation in cerebral cortical cells caused by radiation exposure would lead to impairment of at least some of these neurological functions compromising quality of life. Studies in mice have shown significant structural alterations of the cerebral cortex eight weeks after exposure to four 5 Gy fractions of γ radiation [1]. However, long-term follow up *in vivo* data on underlying mechanisms of changes in cerebral cortex after exposure to a clinically relevant dose of γ radiation is not available in the literature.

Radiation exposure is intimately linked to the production of reactive oxygen species (ROS) and due to its high oxygen consumption and metabolic rate, the brain is more susceptible to ROS and oxidative stress than other organs [10]. Increased ROS could react with lipids, DNA, and proteins leading to the generation of more reactive species and the establishment of a state of perpetual oxidative stress in cells compromising cerebral cortex function [11,12]. Brain due to its high lipid content is particularly vulnerable to oxidative stress-induced lipid peroxidation, which not only generates lipid-based free radicals but also produces a number of highly reactive aldehydes such as malondialdehyde and 4-hydroxy-2-nonenal (4-HNE). These reactive aldehydes in turn react with cellular proteins to form adducts, which have been implicated in neurodegenerative diseases including Alzheimer's disease [13]. When produced in excess of cellular anti-oxidant capacity, ROS are known to induce, apart from other damages, DNA double stand breaks (DSB), the most lethal form of DNA damage. Experimental evidence indicates that unrepaired DSB could induce cell death, and misrepaired DSB has the potential of causing genomic instability [14,15]. DNA DSB in non-dividing cells is commonly repaired by non-homologous end joining (NHEJ) and aging has been associated with a decline in Ku70, Ku80, and DNAPKcs which are considered major players of the NHEJ pathway [16-22].

Persistent induction of DNA damage due to sustained ROS production results in a perpetual DNA damage response [23,24]. The tumor suppressor gene p53 due to its pivotal role in DNA damage response such as cell cycle arrest, DNA repair, and cell death induction remains an important player in maintenance of cellular homeostasis and genomic integrity after radiation exposure. Upon radiation-induced ROS generation and ensuing DNA damage, p53 is activated leading to alterations in the level of its downstream effectors such as Bax, Bcl2, and p21 resulting in the induction of apoptosis and growth arrest [25]. p53, which is mutated in >50% of human cancers, has also been reported to play important roles in aging and increased p53 activity could usher in premature aging [24,26]. However, cellular senescence and aging is a complex process involving multiple signaling pathways [27-30] and association of the tumor suppressors p16^Ink4a^ and p19^Arf^ with senescence is well documented in literature [31,32].

Radiation injury to the brain has been shown to upregulate intermediate filament proteins such as nestin and glial fibrillary acidic protein (GFAP), which are also reported to be associated with oxidative stress, aging, and neurodegeneration [33,34]. The intermediate filament proteins nestin and vimentin are associated with the developing central nervous system (CNS) and upon terminal differentiation of neural precursor cells to astrocytes and neurons, nestin is no longer expressed and is substituted by GFAP and vimentin [35]. Re-expression of embryonic proteins such as nestin and upregulation of GFAP, which reflects proliferative activation of astroglial cells have been observed in radiation-induced CNS injury and increased cellular stress in brain [12,35-41].

Radiation environment in outer space, compared to that on earth, mostly consists of high-energy protons and heavy ions such as ^56^Fe, ^28^Si, ^16^O, and ^12^C and associated secondary particle radiation [42-44]. While solar particle events (SPE) with mostly proton radiation are sporadic, the galactic cosmic radiation (GCR) with most of its dose equivalent contributed by heavy ion radiation is ambient is space. Heavy ion radiation with high linear energy transfer (high-LET - deposits more energy per unit volume of tissue compared to low-LET γ radiation prevalent on earth) characteristics is vastly more damaging compared to proton and γ radiation not only due to its densely ionizing primary track but also due to the greater number of highly ionizing secondary delta ray tracks [45-47]. Consequently, from astronauts' health point of view, a major concern for the National Aeronautics and Space Administration (NASA) during long duration space missions is exposure to heavy ion radiation and its consequences on the CNS. Additionally, heavy ion radiation therapy of brain tumors has been shown to inflict subsequent neurological deficits such as cognitive and memory loss that are predicted to be in part due to alterations in cerebral cortex [48-50]. Also, exposure to high-LET neutron radiation has been shown to induce hypoplasia of the cerebral cortex in the developing mouse brain [51]. While most of the animal studies involving high-LET radiation focused on hippocampus [11,52-58], very few have dealt with cerebral cortex and fewer have reported underlying mechanisms. The current study undertakes long-term follow up investigations of molecular events in cerebral cortex associated with γ radiation exposure and how these events relate to heavy ion radiation exposure. We have shown that there were increased oxidative stress and accelerated senescence signaling after 2 Gy γ radiation. Importantly, we also showed that the effects of oxidative stress and accelerated aging were more pronounced in mice exposed to heavy ions compared to γ radiation.

## RESULTS

### Persistently raised levels of ROS in cerebral cortical cells after radiation exposure

**Figure 1:**
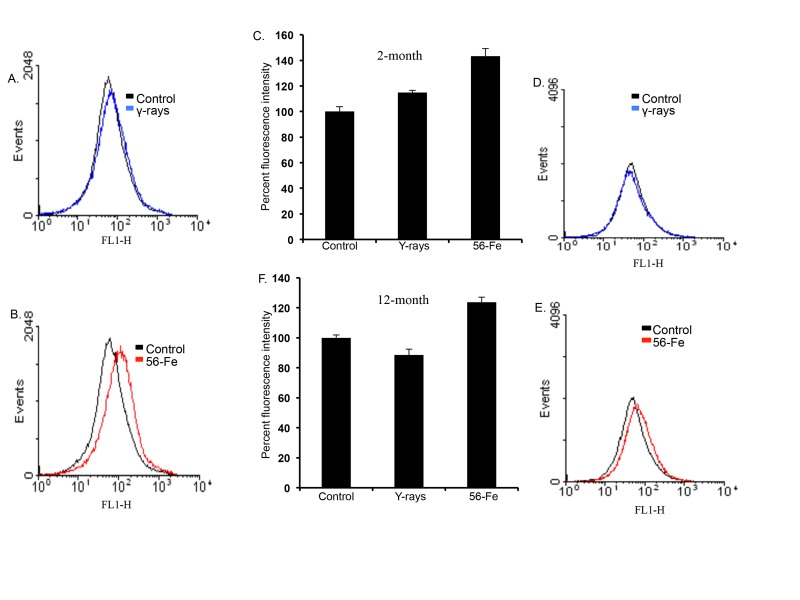
Increased reactive oxygen species (ROS) in cerebral cortical cells after ^56^Fe radiation. (**A**) Flow cytometry histogram showing ROS level two months after γ radiation. (**B**) Flow cytometry histogram showing ROS level two months after ^56^Fe radiation. (**C**) Quantification of ROS level two months after radiation presented as mean ± standard error of mean (SEM). (**D**) Flow cytometry histogram showing ROS level twelve months after γ radiation. (**E**) Flow cytometry histogram showing ROS level twelve months after ^56^Fe radiation. (**F**) Quantification of ROS level twelve months after radiation presented as mean ± SEM.

ROS levels were distinctly increased in freshly isolated cerebral cortical cells two and twelve months after exposure to 1.6 Gy (equitoxic to 2 Gy γ radiation; [59]) of ^56^Fe radiation compared to shams and 2 Gy γ-irradiated samples. (Figure 1A, B, D, and E). Quantification of flow cytometry data demonstrates a significant increase in ROS levels after ^56^Fe exposure at both time points relative to control and γ radiation (for 2-month post-radiation: p<0.008 compared to control and p<0.05 compared to γ radiation; for 12-month post-radiation: p<0.04 compared to control and p<0.01 compared to γ radiation; Figure 1C and F). There was a statistically significant increase in ROS levels two month after γ radiation (Figure 1A and C; p<0.03 compared to control). We did not observe any detectable alterations in ROS levels in twelve-month post-γ-irradiation groups relative to controls (Figure 1D and F).

### Increased lipid peroxidation, oxidative DNA damage and apoptosis after radiation exposure was associated with a decrease in cortical thickness

**Figure 2:**
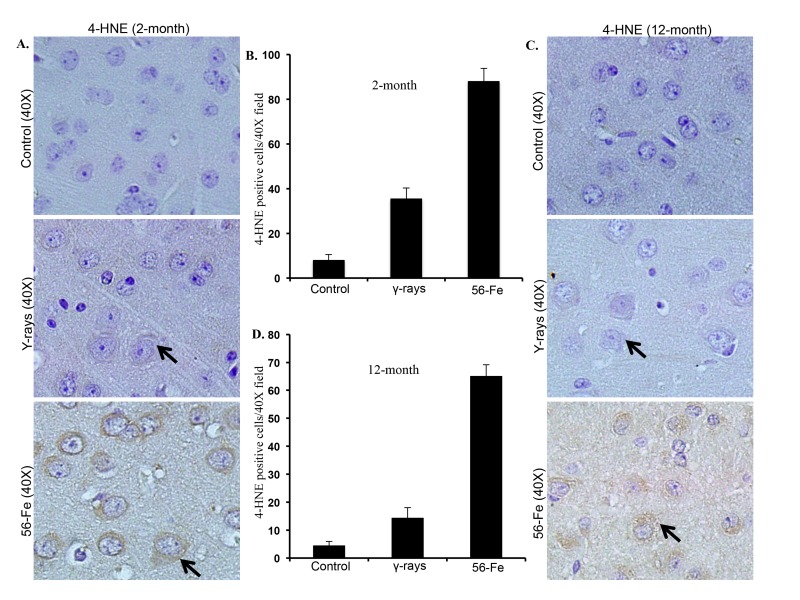
Lipid peroxidation in cerebral cortex was greater after ^56^Fe radiation. **(A**) Immunohistochemical staining (arrow) of cerebral cortex for 4-hydroxy-2-nonenal (4-HNE) two months after radiation. (**B**) Quantification of 4-HNE staining two months after exposure presented as mean ± SEM. (**C**) Immunohistochemical staining (arrow) for 4-HNE twelve months after radiation. (**D**) Quantification of 4-HNE staining twelve months after radiation presented as mean ± SEM.

Persistently elevated levels of 4-HNE indicated by brown coloration visible in the cytoplasm around nuclei both two and twelve months after ^56^Fe radiation were observed (Figure 2A and C). Although less than ^56^Fe radiation, we also observed increased 4-HNE levels after γ radiation (Figure 2A and C). Quantification of 4-HNE staining showed significantly more staining in two as well as twelve months post-^56^Fe irradiation samples relative to γ radiation and controls (for 2-month p<0.0000001 compared to control and p<0.0006 compared to γ radiation; for 12-month p<0.00000006 compared to control and p<0.000006 compared to γ radiation; Figure 2B and D). There was also a significant increase in 4-HNE staining two- and twelve-month post-γ-irradiation relative to controls (for 2-month p<0.00003 and for 12-month p<0.02; Figure 2B and D).

**Figure 3:**
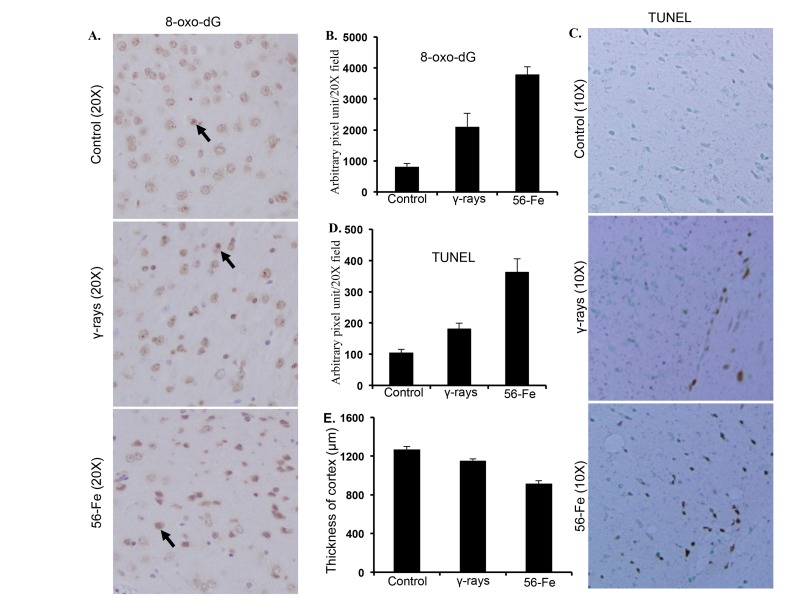
Assessing oxidative DNA damage and cell death in cerebral cortex twelve months after radiation. (**A**) Immunohistochemical staining of cerebral cortex for 8-oxo-dG after exposure to γ and ^56^Fe radiation. (**B**) Quantification of 8-oxo-dG staining in cerebral cortex presented as mean ± SEM. (**C**) TUNEL staining of cerebral cortex after exposure to γ and ^56^Fe radiation. (**D**) Quantification of TUNEL staining of cerebral cortex after exposure to γ and ^56^Fe radiation presented as mean ± SEM. (**E**) Measurement of cerebral cortex thickness in H&E stained histological sections presented as mean ± SEM.

Markedly increased 8-oxo-dG staining in cerebral cortex twelve months after irradiation was observed (Figure 3A). Quantification showed significant difference in 8-oxo-dG staining between γ and ^56^Fe-irradiated samples (for ^56^Fe radiation p<0.0001 compared to control and p<0.04 compared to γ radiation; Figure 3B). Although less than ^56^Fe radiation, we also observed significantly more 8-oxo-dG staining after γ radiation compared to control (p<0.001; Figure 3A and B). TUNEL stain indicating cell death also showed greater number of positive cells twelve months after ^56^Fe radiation compared to γ radiation and quantification showed significant difference between the two types of radiation (for ^56^Fe radiation p<0.0003 compared to control and p<0.01 compared to γ radiation; Figure 3C and D). Small but significant increase in TUNEL positive cells was also observed after γ radiation relative to control (p<0.01; Figure 3C. Measurements of cortical thickness showed significantly greater decrease twelve months after exposure to ^56^Fe radiation relative to control and γ radiation (p<0.00001 compared to control and p<0.0002 compared to γ radiation; Figure 3E). Cortical thickness twelve months after γ radiation was also decreased which was statistically significant relative to control (p<0.008; Figure 3E). Volumetric measurement using magnetic resonance imaging (MRI) also showed decreased brain volume twelve months after radiation exposure (Supplementary figure 1A). While γ radiation showed small but statistically significant decrease in brain volume ((p<0.05 compared to control), the ^56^Fe radiation showed greater decrease than γ radiation (p<0.04 compared to control and γ radiation; Supplementary materials and methods and Supplementary figure 1A). Physical activity assay performed using a barrier (12″ in length and width and 3″ in height) showed irradiated mice taking significantly more time to climb the barrier relative to controls and ^56^Fe irradiated mice needed the most time (for γ radiation p<0.004 compared to control and for ^56^Fe radiation p<0.01 compared to control and γ radiation; Supplementary materials and methods and Supplementary figure 1B). Additionally, γ irradiated mice also showed increased time required climbing the barrier relative to controls (p<0.02 compared to control; Supplementary figure 1B).

### Exposure to radiation decreased DNA repair proteins and increased DNA damage response and senescence markers in cerebral cortex

**Figure 4:**
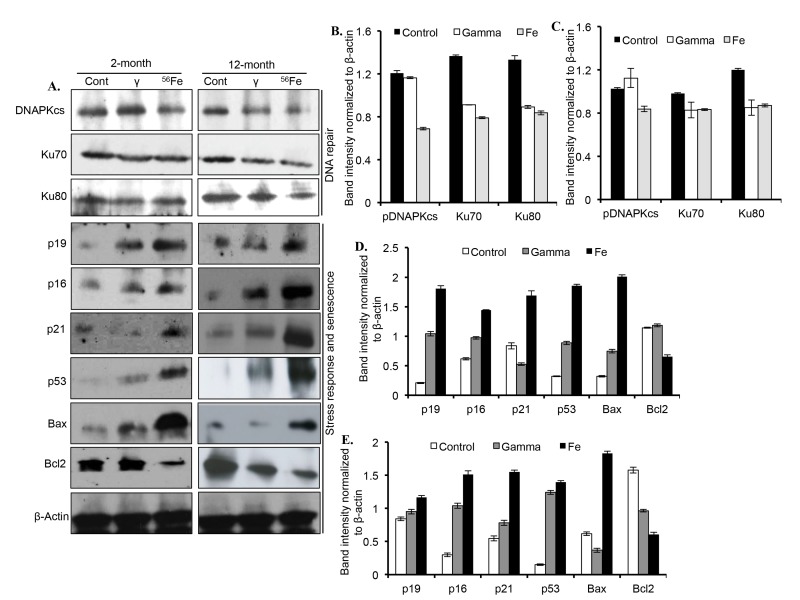
Assessing DNA repair and senescence markers in cerebral cortex. (**A**) Immunoblot images of DNA double strand break repair (Ku70, Ku80, and DNAPKcs), and senescence and DNA damage response (p19, p16, p21, p53, Bax, and Bcl2) proteins two and twelve months after radiation. (**B**) Quantification of Ku70, Ku80, and DNAPKcs two months after radiation. (**C**) Quantification of Ku70, Ku80, and DNAPKcs twelve months after radiation. (**D**) Quantification of p19, p16, p21, p53, Bax, and Bcl2 two months after radiation. (**E**) Quantification of p19, p16, p21, p53, Bax, and Bcl2 twelve months after radiation. Quantification data (panel **B** to **E**) is presented as mean ± SEM.

Proteins involved in DNA DSB repair, DNA damage response, and senescence were markedly altered two and twelve months after radiation exposure (Figure 4A). Quantitatively, a significant decrease in DNAPKcs level, relative to control and γ radiation, was observed after ^56^Fe radiation at both the time points (Figure 4B and C). Compared to control, Ku70 levels were significantly decreased two months after γ and ^56^Fe radiation. However, compared to γ radiation, there was greater decrease in Ku70 levels after ^56^Fe radiation (Figure 4B and C). Although, compared to control, Ku70 was decreased significantly in both the radiation types, its levels were similar in γ and ^56^Fe radiation at the twelve-month time point (Figure 4B and C).

Compared to controls, the levels of Ku80 were reduced in both the radiation types two and twelve months after exposure. (Figure 4B and C). At both the time points, however, the levels of Ku80 were not different between the two radiation types (Figure 4B and C). While compared to controls the levels of cell-cycle arrest and DNA damage response marker proteins p19, p16, and p53 were noticeably increased two and twelve months after γ radiation, the levels of these proteins after ^56^Fe radiation showed greater increase than γ radiation (Figure 4D and E). Although p21 is decreased at two months after γ radiation, its level was increased at twelve months post-exposure relative to controls. Bax level was increased at two months but decreased at twelve months after γ radiation. While Bcl2 level two months after γ radiation was similar to control, its level relative to control was lowered at twelve months after γ radiation (Figure 4D and E). However, after ^56^Fe radiation at both the time points p21 and Bax were increased and Bcl2 was decreased relative to control and γ radiation (Figure 4D and E).

### Reactive gliosis was associated with re-expression of nestin and upregulation of GFAP

**Figure 5:**
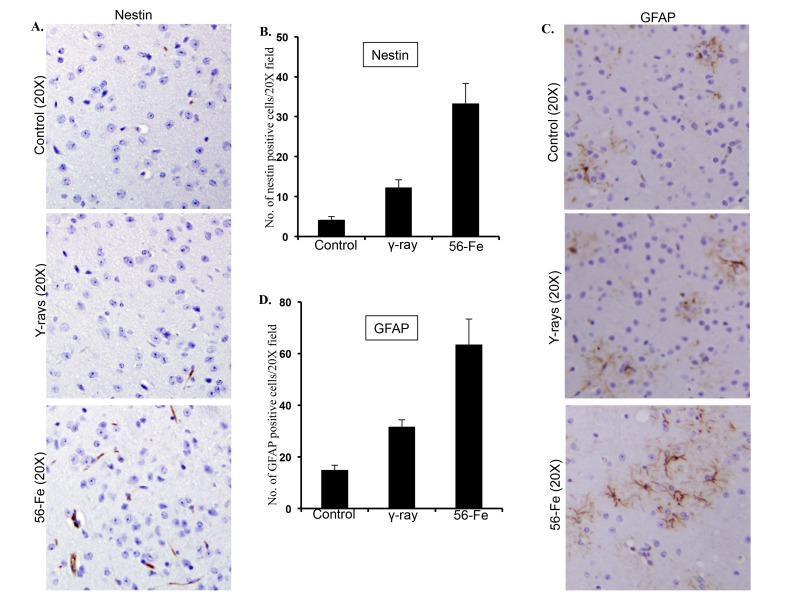
Assessing reactive gliosis twelve months after radiation exposure. (**A**) Comparing immunohistochemical staining of nestin in cerebral cortex after radiation. (**B**) Quantification of nestin staining in cerebral cortex presented as mean ± SEM. (**C**) Comparing immunohistochemical staining of GFAP in cerebral cortex after radiation. (**D**) Quantification of GFAP staining in cerebral cortex presented as mean ± SEM.

Stressful stimuli in brain are associated with activation of astroglial cells resulting in re-expression of nestin and upregulation of GFAP [60]. Our observations showed that there was a significant enhancement of nestin staining in the twelve-month post-^56^Fe radiation exposure samples (for ^56^Fe radiation p<0.0007 compared to control and p<0.003 compared to γ radiation; Figure 5A and B). Also, significantly higher expression of GFAP was observed in cerebral cortex twelve months after ^56^Fe irradiation (for ^56^Fe radiation p<0.02 compared to control and p<0.05 compared to γ radiation; Figure 5C and D). Compared to control, statistically significant increase in nestin (p<0.01; Figure 5A and B) and GFAP (p<0.002; Figure 5C and D) staining was also observed twelve months after γ radiation exposure.

## DISCUSSION

Radiation exposure on one hand has been reported to induce long-term changes in CNS resulting in functional impairments such as motor and sensory disturbances, and learning and memory deficits [61,62] and on the other it has also been associated with risk of initiation and promotion of brain tumor [7,8,63]. However, we still lack a clear mechanistic understanding required to comprehend the persistent molecular events, which unfold in the brain after radiation exposure and has the potential to cause overt neurological deficits or tumor later in life. Importantly, due to its structural makeup and functional demands, the brain is vulnerable to oxidative stress and persistent oxidative stress has been implicated in neurodegeneration and cancer [64]. Although there are reports relating radiation, both γ and ^56^Fe, to changes in the cellular redox status in brain, most of the studies are relatively short term [65-68], very few have involved cerebral cortex, and fewer had long-term follow up investigation at the molecular level in the cerebral cortex. Here we demonstrated that, relative to control, there was persistently higher ROS production after γ radiation. We also observed that persistent ROS production was markedly more after ^56^Fe radiation relative to control and γ irradiation in cerebral cortical cells. While we observed increased lipid peroxidation, DNA damage, and apoptosis along with decreased cortical thickness and brain volume after γ radiation, these effects were more prominent after ^56^Fe radiation. We also demonstrated that there was increased p21, p19, and p16, and decreased DNA repair proteins in cerebral cortex after radiation exposure suggesting premature senescence.

Radiation-induced damage to biomolecules such as proteins and DNA has mostly been attributed to generation of ROS and consequent oxidative stress, which has been implicated in neurodegeneration, aging, and cancer [64,69-71]. Indeed, oxidative stress associated modifications of biomolecules are known hallmarks of the aging brain [11,72] and it is expected that heavy ion radiation with its propensity to cause higher oxidative stress and increased damage to biomolecules could accelerate changes in brain commonly associated with aging. Our results of ROS measurement in cerebral cortical cells confirm the notion that heavy ion radiation induces higher oxidative stress relative to γ radiation and are consistent with our earlier results in intestinal epithelial cells [73]. Importantly, chronic oxidative stress leading to sustained DNA damage and continued activation of p53 dependent DNA damage response has been reported to induce chronic elevation of p21 level that could result in continued growth arrest leading to cellular senescence and short life span [24,74,75,75-78]. Indeed, our immunoblot results showed increased p53 and consequent increase in p21 suggesting senescence of cortical cells after radiation exposure. Interestingly, while persistent elevation of p21 is induced by oxidative stress-mediated DNA damage response, its sustained higher level is also known to result in the activation of signaling events promoting mitochondrial perturbation and increased ROS production [79]. Persistent elevation of p53 in γ as well as in ^56^Fe-irradiated mice also illustrates a state of perpetual DNA damage response (DDR), which is supported not only by elevated p21 but also by alterations of other p53 effectors such as Bax and Bcl2. Our observations of increased Bax and decreased Bcl2 in irradiated mice led us to believe that the balance of pro- and anti-apoptotic factors in cerebral cortex is in favor of cell death. It has been reported that with advancing age there is a gradual decline in normal brain volume and in neurodegenerative diseases a marked cerebral atrophy with decline in cognitive function have been observed [80,81]. Reduction of cortical thickness, a hallmark of degenerative changes, observed after radiation exposure is probably due to cell death (confirmed by our TUNEL assay) mediated by p53-induced alterations of Bax and Bcl2 levels. Taken together our results in this study demonstrated that radiation exposure caused chronic oxidative stress, persistent oxidative DNA damage, and such DNA damages were associated with increased DDR leading to growth arrest as well as cell death in cerebral cortex. Considering the fact that ROS generated immediately after radiation are due to radiolysis of water molecules and are short lived, we believe that the persistent oxidative stress in cerebral cortex observed in our study is due to metabolically regenerated ROS of which mitochondria are the major source [82]. Oxidative stress-associated damage to biomolecules such as oxidative proteins and DNA damage has been implicated in a decline in functional competence of cerebral cortex [11,72,83]. Consequently, our results in cerebral cortex demonstrated a potential risk of developing long-term functional deficit as well as cellular transformation after radiation exposure.

Effects on CNS from heavy ion space radiation exposure due to its higher damaging capacity than γ radiation is a major health concern for astronauts undertaking long duration space missions. Apprehension about acute and long-term heavy ion radiation exposure risks to CNS is primarily due to lack of *in vivo* data in humans and animal models. However, in recent years, substantial work has been published which sheds lights on the effects of heavy ion radiation on different parts of the brain [11,52-58,84,85]. Evidence based on *in vivo* and *in vitro* studies has demonstrated that high-LET radiation adversely alters spatial learning, cognitive, memory, and other CNS faculties [11,54,55,85-88]. Our results also suggest that heavy ion space radiation with higher oxidative stress and greater associated changes has elevated risk of developing neuronal deficit and transformation and will require additional studies.

Radiation-induced persistently increased ROS and DNA damage could lead to sustained cell-cycle arrest potentially leading to senescence [89] and the cell's capability to repair DNA damage including DSB have been reported to decline with advancing age [17,19-21]. Additionally, decreased DSB repair proteins has been associated with enhanced cellular senescence and non-functional DSB repair proteins are known to accelerate organismal aging [16-22]. Our results showing decreased Ku70, Ku80, and DNAPKcs proteins in cerebral cortex, we speculate, is due to sustained higher oxidative stress, which could affect transcription, translation, and stability of repair proteins. Decreased repair proteins leading to reduction in DNA repair, we believe, would accelerate the onset of aging after ^56^Fe radiation [90]. Additionally, cellular stress has also been reported to consistently upregulate two other well-characterized aging markers, p19^Arf^ and p16^Ink4a^, heralding stress-induced senescence. Importantly, p19^Arf^ is activated in cells under stress including radiation exposure and is associated with the augmentation of p53-dependent DNA damage response through sequestration of MDM2 and thus stabilization of p53 [91]. Indeed, marked upregulation of p19^Arf^ after both γ and ^56^Fe radiation may have played a prominent role in enhanced p53-dependent DNA damage response in our experimental setting to affect cellular senescence. Although the regulation of p16^Ink4a^, which is a cyclin-dependent kinase inhibitor and is progressively upregulated with advancing age, is not well understood [32], chronic upregulation of both p19^Arf^ as well as p16^Ink4a^ further confirms cellular senescence in cerebral cortex after radiation exposure. Additionally, decreased brain volume in MRI and increased time required to climb the physical barrier after radiation exposure provided further evidence linking premature senescence and radiation (Supplementary figure 1A and B). However, additional functional studies will be required to relate observed changes in cerebral cortex after radiation to alterations in spatial learning, cognition, memory, and other CNS faculties of the organism.

**Figure 6:**
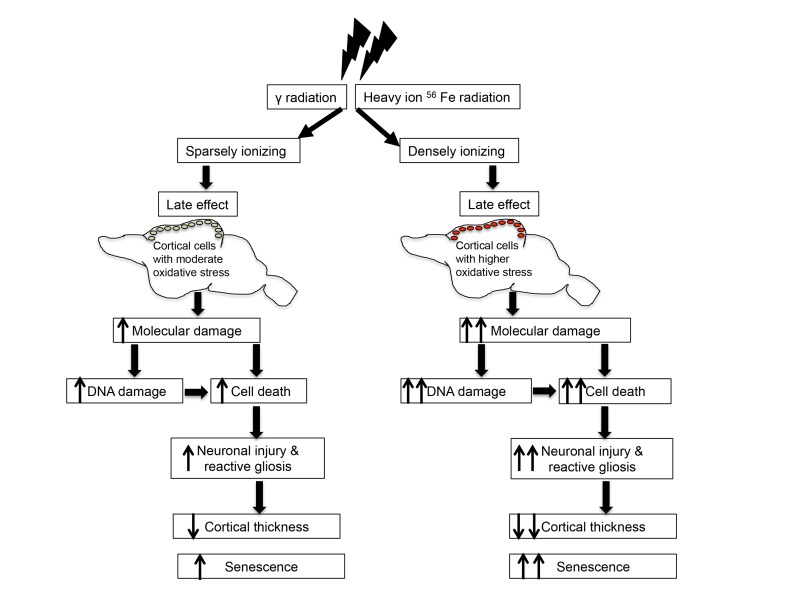
Schematic overview of radiation-induced chronic oxidative stress and accelerated aging.

Radiation-induced cellular damage in brain not only cause enhanced DNA damage response and senescence, but it also triggers repair and remodeling process through a proliferative response known as reactive gliosis [35,36]. Our results are consistent with previous reports relating nestin and GFAP to both γ and heavy ion radiation exposure [36,84]. We demonstrated that these two embryonic proteins showed higher expression in response to both γ and heavy ion radiation exposure suggesting activation of astroglial cells in cerebral cortex. Upregulation of both nestin and GFAP after radiation support a state of reactive gliosis in cerebral cortex. However, reactive gliosis denoted by increased nestin and GFAP does not support regeneration of neurons but instead leads to glial scar formation [36,84], which along with apoptosis may have aggravated shrinkage of cerebral cortex after radiation exposure. Glial scar formation rather than neuronal regeneration is further supported by the upregulation of GFAP that indicates that radiation-induced activation of astroglial proliferative response in cerebral cortex is committed to astrocytosis and subsequent scar tissue formation [84]. Our study has provided evidence that radiation exposure in the mouse brain accelerates the appearance of biological indicators of aging at the molecular level (Figure 6). Knowledge of adverse long-term sequelae of radiation exposure on cerebral cortex will not only allow us to assess risk but will also permit us to design strategies to minimize the effects of radiation on normal tissues.

## METHODS

### Ethics statement on mice

Six to eight weeks old female C57BL/6J mice were purchased from Jackson Laboratories (Bar Harbor, ME). Mice were housed at the Georgetown University (GU) and Brookhaven National Laboratory (BNL) animal care facilities. Both the facilities are Association for Assessment and Accreditation of Laboratory and Animal Care International (AAALACI)-accredited and all animal procedures were performed as per protocols approved by the Institutional Animal Care and Use Committees (IACUC) at the GU and at the BNL. Mice were housed in groups of five in autoclaved cages and bedding materials in a separate room with 12-h dark and light cycle maintained at 22 °C in 50% humidity. All animals were provided certified rodent diet (LabDiet #5053, Brentwood, MO) with filtered water *ad libitum* and CO_2_ asphyxiation was used for euthanasia. Our research followed Guide for the Care and Use of Laboratory Animals, prepared by the Institute of Laboratory Animal Resources, National Research Council, and U.S. National Academy of Sciences. Investigation has been conducted in accordance with the ethical standards and according to the Declaration of Helsinki and according to national and international guidelines and has been approved by the authors' institutional review board.

### Radiation

Exposure to heavy ion radiation (^56^Fe; energy: 1GeV/nucleon; dose rate: 1 Gy/min) was performed at the NASA Space Radiation Laboratory (NSRL) at BNL and ^137^Cs was used as a source of γ radiation (dose rate: 1 Gy/min) and control mice were sham irradiated. During radiation exposure mice (n=15) were placed in small transparent plastic boxes (3″×1.5″×1.5″) with holes for ventilation. Mice were exposed either to 1.6 Gy of ^56^Fe or to 2 Gy of γ radiation and irradiation experiments was performed three times. The ^56^Fe radiation dose is equitoxic to γ radiation and was calculated using a relative biological effectiveness (RBE) factor of 1.25 determined earlier [59]. For ^56^Fe irradiation, the NSRL beam physics team performed the dosimetry, dose rate, and beam uniformity and mice were exposed to constant LET by placing them at the entrance plateau region of the Bregg peak [92-95]. Mice were shipped directly from the vendor to BNL one week prior to radiation and on the day after irradiation all the mice were shipped in a temperature controlled environment to GU for a same day delivery. Mice were followed for up to twelve months, brain surgically removed at two and twelve months post-exposure, washed and cleaned in phosphate buffered saline (PBS), two hemispheres separated in the middle, and one half fixed in 10% buffered formalin. While immersed in sterile PBS, multiple coronal sections of the other half of the brain were made and the cerebral cortex was separated from the rest of the brain under a dissecting scope (MZ6, Leica Microsystem, Wetzlar, Germany) as per protocol described earlier [96]. Cerebral cortex was either used for preparation of cortical single cell suspension for measuring reactive oxygen species (ROS) or flash frozen in liquid N_2_ and stored at −80 °C for immunoblots.

### Measuring ROS in cerebral cortical cells

Separated cerebral cortex (n=5 mice per group) was mechanically dissociated into single cell suspensions in Hank's balanced salt solution (HBSS) and filtered through a sterile 70 μm nylon mesh (BD Biosciences, Sparks, MD) as per protocol described previously [1,97]. Cells were centrifuged (200xg) for 10 min at room temperature (RT) and supernatant discarded. Cell pellet was resuspended in 1 ml of PBS at RT. A 2 mM solution of H2DCFDA (Invitrogen, Carlsbad, CA) was freshly prepared in ethanol and 5 μl were added to the cell suspension (final concentration 10 μM) and incubated at 37 °C for 20 min. Cells were centrifuged at 200xg for 5 min, supernatant discarded, and cell pellet resuspended in 500 μl of PBS. Flow cytometric analysis was performed in duplicate using FACSCalibur (BD Biosciences) and acquired data were analyzed using WinMDI v2.8 software.

### Immunoblot analysis

Immunoblots were performed using standardized protocol. Flash-frozen cerebral cortical samples isolated two and twelve months after exposure to 2 Gy γ radiation and equitoxic 1.6 Gy of ^56^Fe radiation were used for immunoblots. Cortical tissues from 5 mice in each group were pooled and homogenized in ice cold lysis buffer (0.5% sodium deoxycholate; 0.5% NP-40; 10mM EDTA in PBS and protease inhibitor cocktail (Sigma, St. Louis, MO)), centrifuged at 12000xg at 4 °C for 15 min, and protein concentration was estimated in the supernatant by the Bradford method. Equal amount proteins with appropriate volume of loading buffer were loaded onto SDS-PAGE. Separated proteins were transferred to polyvinylidene fluoride (PVDF) membrane, treated with 5% non-fat milk in tris-buffered saline with 0.1% Tween (TBST), and exposed to specific primary antibodies for DNAPKcs (Sc-5282; diltion-1:200; Santa Cruz Biotechnology, Santa Cruz, CA), Ku70 (Sc-17789; diltion-1:200; Santa Cruz Biotechnology), Ku80 (Sc-9034; diltion-1:400; Santa Cruz Biotechnology), p53 (Sc-99; diltion-1:500; Santa Cruz Biotechnology), Bax (Sc-7480; diltion-1:250; Santa Cruz Biotechnology), Bcl2 (Sc-7382; diltion-1:250; Santa Cruz Biotechnology), p21 (Sc-6246; diltion-1:500; Santa Cruz Biotechnology), p16 (Sc-1661; diltion-1:200; Santa Cruz Biotechnology), p19 (07-543; diltion-1:200; EMD Millipore, Billerica, MA), and β-actin (Sc-47778; diltion-1:2000; Santa Cruz Biotechnology) and developed by chemiluminescence (Thermo Scientific, Rockford, IL) detection system. Results were recorded by autoradiography, images scanned and displayed. We used ImageJ v1.46 software for densitometric quantification of immunoblot images and band intensity of each protein normalized to β-actin is presented in bar graphs.

### Cerebral cortex histology

Twelve months post-irradiation brain samples were fixed in 10% buffered formalin for 72 hours, paraffin embedded, and 4 μm sagittal sections were obtained for further processing. For histologic analysis, slides were stained with hematoxylin and eosin (H&E) using a standard protocol and visualized by bright field microscopy at 4X microscopic magnification. Cortical thickness was measured using a protocol described previously [1]. Briefly, slide images were captured with an Olympus DP70 camera on an Olympus BX61 microscope and the thickness of the cerebral cortex was measured using ImageScope (Aperio Technologies, Vista, CA) software and results expressed in μm. For each study group, sections from seven mice were measured and three sections from each mouse were used. Unstained sections were used for terminal deoxynucleotidyl transferase dUTP nick end labeling (TUNEL) assay and immunohistochemistry.

### TUNEL assay

TUNEL assay on brain sections (6 sections from 6 separate mouse from each group) was performed using ApopTag plus peroxidase in situ apoptosis detection kit (Millipore, Billerica, MA) as per manufacturer's instruction. Stained sections were visualized under bright field microscopy at 20X magnification and twelve random fields per group were captured for analysis.

### Immunohistochemistry

Anti-8-oxo-dG antibody (clone 2E2; 4354-MC-050) was purchased from Trevigen (Gaithersburg, MD) and immunostaining was performed using recommended protocol. Briefly, following incubation with primary antibody overnight at 4 °C and necessary washing steps, sections were incubated with HRP-conjugated secondary antibody for 30 min at room temperature [73]. Diaminobenzidine (*DAB*) detection system (Invitrogen, Carlsbad, CA) was used to visualize staining. Bright field microscopy was used to capture the images of the cerebral cortex at 10X magnification and twelve images from randomly selected visual fields were captured from each group. For nestin, GFAP, and 4-HNE, immunostaining was performed as per protocol described previously [73,98,99]. Briefly, sections were deparaffinized, antigen retrieval performed, endogenous peroxidase activity quenched, and incubated in blocking buffer (0.1% bovine serum albumin in PBS). Sections were then exposed to anti-nestin (Sc-23927; dilution-1:100; Santa Cruz Biotechnology), anti-GFAP (PA5-16291; dilution-1:150; Thermo Scientific) and anti-4-HNE (Ab-46545; 1:200; Abcam, Cambridge, MA) antibodies for 1.5 hr at RT. Signal detection and color development was performed using SuperPicture™ 3^rd^ Gen IHC detection kit (Cat# 87-9673; Invitrogen) and slides were mounted and visualized under bright field microscopy and twelve images from randomly selected visual fields were captured from each group for quantification. Six slides from six mice in each group were stained for each protein and a representative image from one animal is presented in results.

### Data Analysis and statistics

Images were analyzed for TUNEL, 8-oxo-dG, 4-HNE, nestin, and GFAP positive cells using color deconvolution and Image-based Tool for Counting Nuclei (ITCN) plug-ins of ImageJ v1.46 software as per protocol described earlier [73,100,101]. Average number of TUNEL (20X), 8-oxo-dG (20X), nestin (20X), and GFAP (20X) positive nuclei per visual field is presented graphically. Data is presented as mean ± standard error of mean (SEM) and statistical significance between two groups was determined by student's t-test and p<0.05 was considered significance.

## Supplementary methods

### Activity tests

Mice (n=5 mice per study group) were irradiated with 2 Gy γ or 1.6 Gy of ^56^Fe radiation and twelve months after irradiation each mouse were placed inside a barrier (12″ in length and width and 3″ in height) and time taken to come out of the barrier was noted in seconds. Results are presented as average time (sec) per mouse relative to sham-irradiated controls.

### Mouse brain magnetic resonance imaging (MRI)

The MRI was performed on a horizontal Bruker 7T spectrometer with a 20 cm bore run by Paravision 4.0 software as per protocol standardized in the Preclinical Imaging Research Laboratory at the Lombardi Comprehensive Cancer Center at the Georgetown University. Mice (n=3) were anesthetized and anesthesia maintained with 1.5% isoflurane, 30% oxygen and 70% nitrous oxide and animals were placed on a custom-made stereotaxic animal holder equipped with temperature and respiration monitoring and imaged in a 35 mm birdcage radiofrequency coil. The sequence used to assess brain volume was a 3D RARE, matrix: 256×256×256, RARE factor: 8, TR: 500 ms, TE: 7.45 ms, FOV: 3 cm. Volumetric measurement of the brain was determined from MRI stacked images using the Measure Stack plugin tool (developed by Dougherty RF and available at http://www.optinav.com/imagej.html) of the ImageJ v1.46 software as per developer's instruction.


