# Supplementary material for: Therapeutic and space radiation exposure of mouse brain causes impaired DNA repair response and premature senescence by chronic oxidant production
Source: Aging (Albany NY). 2013 Aug 6;5(8):607–22. doi: 10.18632/aging.100587 (PMC3796214; doi:10.18632/aging.100587)
Supplement: Supplementary file 1 [file aging-05-607-s001.pdf]

SUPPLEMENTARY FIGURE

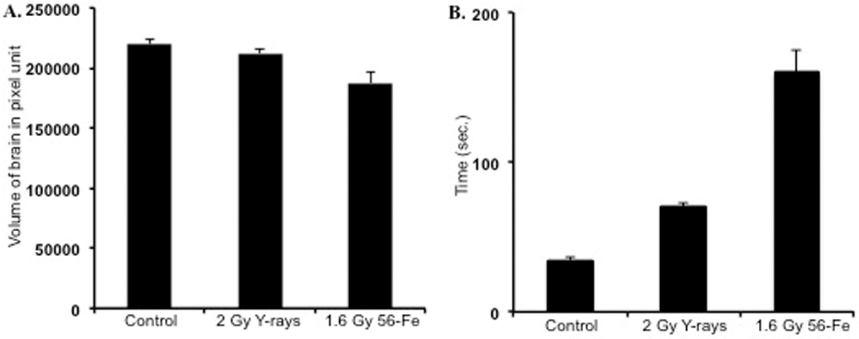

Supplementary Figure 1. Radiation exposure caused decreased brain volume and increased time to cross a barrier. A) Volumetric measurement of brain performed using magnetic resonance imaging (MRI) showed decrease brain volume after radiation exposure. Brain volume was decreased more after ^{56}Fe radiation. B) Activity experiment performed using a physical barrier showed increased time required by the irradiated mice to climb the barrier. ^{56}Fe irradiated mice needed more time to come out of the barrier. Data presented as mean ± SEM.
